# Assessing content and factors influencing responses to information requests in community pharmacies in Jordan: A simulated patients study

**DOI:** 10.1371/journal.pone.0264224

**Published:** 2022-02-18

**Authors:** Eman A. Hammad, Sinaa Al-Aqeel, Eman Elayah, Deema Jaber

**Affiliations:** 1 Department of Biopharmaceutics and Clinical Pharmacy, School of Pharmacy, University of Jordan, Amman, Jordan; 2 Clinical Pharmacy Department, College of Pharmacy, King Saud University, Riyadh, Kingdom of Saudi Arabia; 3 Department of Clinical Pharmacy, Faculty of Pharmacy, Zarqa University, Zarqa, Jordan; University of South Australia, AUSTRALIA

## Abstract

**Objectives:**

To assess the management of requests for information about a prescription only medicine (simvastatin for treatment of dyslipidemia) by pharmacy staff in community settings and explore the factors influencing the information content.

**Methods:**

A cross sectional study conducted using the stimulated patient (SP) method between November 2018 and May 2019. The SP conveyed the request at the beginning of the encounter in a standardized way based on predetermined plots and was instructed to ask the pharmacy staff directly if information was not discussed spontaneously. After the visit, the SP provided written feedback including information about the scenario and a copy of individualized feedback. The study was reported according to the checklist for reporting research using simulated patient methodology (CRiSP). Factors influencing information content with or without information demand were investigated.

**Results:**

A total of 55 visits were analyzed. The average content score for the information discussed spontaneously was 16.2% with the standard deviation (SD) equal to15.6. The score improved significantly after information was demanded by the SP; the average total information content score became 34.4% (SD = 16) with p < 0.001. The score of information discussed spontaneously was higher for male pharmacy staff, older age, more experience, and a Pharm D degree. When the SP prompted or demanded for information, older pharmacy staff with more experience and with a college degree scored higher. Independent pharmacies, longer visit durations, and less distraction were associated significantly with higher information scores Pharmacy staff aged 35–39 and those with 6–10 years of experience were significant contributors to spontaneous discussion of information with p values = 0.003 and 0.013, respectively. After the SP demanded information, pharmacy staff with less than 5 years of experience and greater confidence as well as longer visits were positively predicting higher information scores with p values of 0.049, 0.04, and 0.04, respectively.

**Conclusions:**

Information provided by community pharmacists responding to information requests about prescription only medications was found to be suboptimal. Patient requests for information were found to be a positive driver for better information content. Further research of mixed methodologies is required to clarify the factors and motivators enabling information exchange in community settings and to outline true training needs.

## Introduction

Community pharmacies are the most accessible health care venue for patient counseling. With increased access to medications and information, the key roles for community pharmacists in rationalizing medication use are growing [1]. Therefore, gathering and sharing essential information with the patient or caregiver is vital to ensure patient safety and optimize therapy outcomes [2, 3].

Preventable diseases, delayed diagnosis, and limited accessibility to screening programs are placing huge burdens across countries [4, 5]. Therefore, there are growing needs to outline effective strategies that can reach out to the patient successfully [4]. Community pharmacists are well-positioned for patient-centered roles due to their convenient location and extended hours of operation [1, 4]. Hence, it is important to outline ways to support community pharmacists in their roles of changing patients’ life styles, reinforcing medication adherence, simplifying dosage regimens, and communicating with patients to establish the desired therapeutic outcomes [3, 6]. The simulated patient (SP) approach is widely used to assess practice assessment since it captures a spontaneous observation without individuals being aware of being evaluated [7]. It is also useful in enforcing improvements by guiding focused and tailored feedback in natural practice settings [8, 9].

Community pharmacies are abundant and highly accessible in Jordan [10, 11]. According to the Jordan Pharmacists Association (JPA) records, there are 3,780 community pharmacies (either pharmacist-owned or within pharmacy chains) staffed with 7,200 pharmacists. Additionally, there is a total of 8,000 medications in Jordan of which 3,000 are classified as over the counter (OTC) drugs. Nevertheless, there is limited control on drug supply and many prescription only medicines (POM) are supplied without prescriptions except for controlled drugs such as sedatives and tranquilizers [12–14]. There is no practice framework for delivering patient-centered pharmaceutical care and the main role of community pharmacists is to focus on product supply [15]. Patient counseling is not provided routinely or systematically to patients attending independent or chain pharmacies [10, 11].

Counseling content and practice were investigated previously in Jordan using the SP approach, namely in responding to symptom scenarios such as headache, insomnia, and proper inhaler techniques. The practice and content of information were found suboptimal with respect to OTC supply and inhaler counseling [12, 13, 16–18]. The need to enhance practice and underline reasons for insufficiencies have been urged. Additionally, factors that influence the content and practice were left unclear. The aim of this study was to assess the management of a request for information about a POM (simvastatin for treatment of dyslipidemia) by pharmacy staff and to explore the factors affecting the content of information.

## Methods

### Study design

A cross sectional study was conducted using the SP method between November 2018 and May 2019. Prior to visits, an independent research assistant approached a senior representative from a convenient sample of community pharmacies in the capital city of Amman. The sample was selected to reflect different socioeconomic areas of the city and different pharmacy types (independent versus chain). The convenient sampling technique was used because it is cheap, efficient, and simple to implement. An independent research assistant informed a representative from the pharmacy about the study and invited them to take part in the study. Informed written consent was obtained from all participating pharmacies.

Pharmacy staff who did not wish to participate in the study were given a sticker and requested to wear it for the upcoming six months. No information was given about the date and time of the visits, the SP identity, or any details of the scenarios.

The visits were conducted by four trained SPs (two females and two males) who were pharmacy students in their fourth year of study. SPs were trained via a one-day program using mixed approaches including role plays, discussions, and peer reflection. The training was delivered by a senior clinical researcher (EH) and focused on how to enact the scenario, collect data, and complete the study forms. The scenario was piloted in eight pharmacies to enhance authenticity and standardization [19]. The visits were audiotaped to improve the reliability of the data record and report duration of the visits. The audiotaped recordings were checked by the principle investigator to ensure the adherence of the SP to the protocol and validity of the visit assessments. Each pharmacy was visited once by one of the SPs. The study was reported according to the checklist for reporting research using simulated patient methodology, known as CRiSP [20].

Ethical approval was obtained from the Institutional Review Board (IRB) at the University of Jordan Hospital (Ref number: 235/2014-30/09/2014).

### Scenarios and visits

The study scenario was adapted with modifications based on previous research and presented in Table 1 [21–23]. The SP conveyed the request at the beginning of the encounter in a standardized way based on predetermined plots and was instructed to ask the pharmacy staff directly if information was not discussed spontaneously.

**Table 1:** The study scenario.

**Plot**	The SP requested advice on simvastatin (he/ she had a sachet of the tablets to show to the pharmacy staff). The medicine was for his/her mother. The mother was 60 years old, had not previously taken the medicine, and had high cholesterol, but she agreed that she would start the medicine to reduce cholesterol.
***Instructions***	The SP welcomed any questions or counseling the pharmacy staff asked or provided. The SP asked for information when not given spontaneously using the following dialogue: “This is for my mother, who is 60 years old, has not previously taken the medicine, and has high cholesterol. The doctor tested her blood and gave this to her one month ago, yet she is not taking it sometimes. She is not obese and not very comfortable with it.”
	If not discussed spontaneously, the SP asked the following questions:
	◾ What is it for?
	• How long should she use it?
	◾ When should she take it?
	◾ Can you explain why she needs to take this medication as she is thin and not eating meat or fatty chicken?

### Assessments of SP visits

The assessment forms were adapted with modifications based on previous research and face validated with two independent senior researchers and a community pharmacist [7, 8, 13, 16, 18, 21–23]. The study forms were completed immediately outside the pharmacy. Forms are available upon request.

These forms assessed content of information and communication skills. Information about pharmacy type (independent or chain), number of waiting customers, day and time of visit, and gender of pharmacy staff were collected. Privacy of the conversation and distracting factors were assessed by the SP on an ordinal scale: 1 indicating poor to 5 indicating very good [23, 24].

#### Content of information

The SP recorded whether the pharmacy staff discussed the following provision of information: who the medicine was for, indication/benefit, the drug name, strength, dose, and time for administration. Additionally, the SP recorded if medical history, known allergies and side effects, and precautions were discussed. Each provision of information was scored 1 point if the pharmacy staff provided in any format. A score of 100% (12 out of 12) represented that the pharmacy staff discussed all relevant information and provided appropriate responses to SP enquiries. The SP recorded whether the information was provided spontaneously or after prompt or demand by the SP. The SP also recorded if written information and non-pharmacological advice were provided.

#### Communication skills

To assess communication skills, the SP recorded if the pharmacy staff introduced himself/herself, welcomed the patient, explained the need to ask the SP questions, maintained eye contact, checked for questions of concern, used open body language, used lay term language, and offered contact number or access back to the pharmacy.

The SP assessed the confidence of the pharmacy staff that was demonstrated in making recommendations on an ordinal scale: 1 indicating poor to 5 indicating very good. The effectiveness of communication was based on previous literature [23, 24].

### Post visit feedback

After completing the assessment form outside the pharmacy, the SP re-entered the pharmacy and disclosed his/her identity with a thank you package including a souvenir mug with written feedback including information about the scenario and a copy of individualized feedback. The SP discussed the feedback in a non-confrontational way with focus on areas for improvement. The SP collected the following information for pharmacy staff: age, professional status (pharmacist with BSC. Pharm. or Pharm D degree or pharmacy technician) and years of experience in community settings.

Before leaving the pharmacy, the SP supplied the pharmacy staff with a form assessing pharmacy staff views on the SP visits and asked for their perception of whether patients are open for information or in a hurry in real practice, whether the pharmacy staff are too busy to provide information, and whether the pharmacy staff needs training and service reimbursement. The responses were Y/N and on an ordinal scale from 1 = strongly disagree to 5 = strongly agree.

### Statistical analysis

Data were coded and entered into the SPSS © database Version 23 for statistical analysis. Descriptive statistics were reported as mean (± SD), median (range) and frequency n (%). Factors influencing information content with or without information demand or prompt by the SP were investigated using independent or paired sample T test and ANOVA when assumptions for parametric testing were fulfilled. When assumptions were violated, nonparametric tests were performed, namely the Mann-Whitney U test, Spearman correlation, Kruskal-Wallis test, and Wilcoxon signed rank sum test. A p value > 0.05 was considered statistically significant.

Checking for normality was carried out using the Shapiro-Wilk test, with P-value > 0.05 indicating normally distributed continuous variables.

Factors were dissected into pharmacy staff related factors and those related to the SP visit. Factors relating to the pharmacy staff were gender, age, years of experience, pharmacy degree, and pharmacist’s confidence. Those related to the visit were day of the visit, time of the visit, busyness (number of waiting customers), visit duration (min), SP’s gender, privacy, and distraction. Dependent variables were the score for information obtained spontaneously (without SP demand or prompt) and the total score for the information content during the visit. A p value < 0.05 was considered statistically significant, and all tests were two-tailed.

General linear models (GLM) were used to build exploratory models of factors predicting information discussed by the pharmacy staff spontaneously (without SP demand) and a total score for information content during the visit. Assumptions for independence of the covariate and homogeneity of the regression slopes were checked.

## Results

A total of 72 community pharmacies were approached and 63 agreed to participate. The study was piloted in 8 pharmacies and consequently 55 visits were analyzed in this study. Fifty percentage of the pharmacy staff approached were females and mostly aged below 30 years with less than five years of experience in community settings. The majority hold a BSc in Pharmacy. Characteristics of the visited pharmacists are presented in Table 2. The average duration of visits was 1.55 minutes with a range 39 seconds to 5.5 minutes.

**Table 2:** Characteristics of pharmacy staff and visits (N = 55)*.

Pharmacy staff	n (%)
**Gender**	
Female	31 (56.4)
**Age group**	
20–24	12 (21.8)
25–29	22 (40.0)
30–34	9 (16.4)
35–39	6 (10.9)
≥40	6 (10.9)
**Years of experience** *	
1–5 years	29 (53.7)
6–10 years	5 (9.3)
11–15 years	7 (12.9)
>15 years	6 (11.1)
Unspecified	7 (12.9)
**Degree** *	
Pharmacist (BSc.Pharm.)	38 (70.4))
Pharmacist (Pharm D)	5 (9.3)
Pharmacy technician (Diploma)	5 (9.3)
Unspecified	6 (11.1)
**Pharmacy Type**	
Chain	28 (50.9)
**Day of the visit**	
Weekdays	33 (60)
**Time of the visit**	
12:00–8:00 pm	39 (70.9)
**Busyness**	
Quiet (0 customers)	37 (67.3)
Low (1–2 customers waiting)	15 (27.3)
Moderate (3–5 customers waiting)	3 (10.9)
Busy (> 5 customers waiting)	0

*Years of experience and pharmacy staff degree were obtained from the feedback form collected on the next day. One feedback form was not returned.

Content and frequency of information provided spontaneously and after prompt or demand by the SP as well as communication skills are presented in Table 3. The mean (SD) content score of information discussed spontaneously was 16.2% (SD = 15.6). A Wilcoxon Signed-Ranks Test indicated that after prompting and demanding information from the pharmacist who did not provide information spontaneously, scores were statistically significantly higher at 34.4% (SD = 16) with p < 0.001.

**Table 3:** Information content and communication skills (N = 55).

The pharmacy staff discussed	Spontaneously n (%)	After prompt	Total
Who is the medicine for?	13 (23.6)	25 (45.5)	38 (69.1)
Any other medications (POM & OTC) being used	4 (7.2)	6 (10.9)	10 (18.1)
Any known allergies	0	1 (1.8)	1 (1.8)
Indication/Benefit	23 (41.8)	18 (50.9)	41 (74.5)
Scientific name	25 (45.5)	11 (20.0)	36 (65.5)
Strength	8 (14.5)	16 (29.1)	24 (43.6)
Dose	12 (21.8)	25 (45.5)	37 (67.3)
Duration	10 (18.0)	15 (27.3)	25 (45.5)
How to take	18 (32.7)	8 (45.0)	24 (43.6)
Side effects/precautions or warnings	0	1 (1.8)	1 (1.8)
Special directions (e.g. to take it at night)	5 (10)	7 (12.7)	12 (21.8)
Respond to patient concerns (Why do I need to take it, I am thin and do not eat meat or fatty chicken?*)	5 (9)	33 (60)	38 (69.1)
**Communication skills**			
Introduced himself /herself	3 (5.5)		
Welcomed (smiled at) the SP	40 (72.7)		
Explained need for asking questions	6 (10.9)		
Maintained eye contact	51 (92.7)		
Asked if SP had questions/concerns	6 (10.9)		
Used open body language	39 (70.9)		
Showed appropriate listening skills	27 (65.9)		
Checked SP understanding of recommendations	13 (23.6)		
Used appropriate language (no jargons)	47 (85.5)		
Offered contact information for follow up	1 (1.8)		

*Answer in any format was scored 1 point “High cholesterol is not necessarily associated with body weight or eating habits. These pills are good to reduce the bad fat in your blood and not related to the fat in the body. Blood fat is what causes heart problems.”.

With respect to communication skills, the pharmacy staff often maintained eye contact, welcomed the SP, used appropriate language, and used appropriate listening skills. However, they often did not introduce himself/herself, asked questions about the SP’s needs, checked the SP’s understanding, or if he/she had questions or concerns. In only one visit, the SP offered contact information for further follow up if needed. Furthermore, non-pharmaceutical advice was provided in 22 (37%) visits while written information was provided in 12 (21%) using manufacturer recommendations or leaflets.

Median (IQR) for privacy of conversations was good to very good 4 (3), and privacy was rated good to very good in 50 (90.9%) visits. Distraction was rated good to very good in 47 (85.5%) visits. Median (IQR) for confidence was 4 (3), and the SP rated confidence good to very good in 51 (92.7%) visits.

### Factors contributing to content of information

Table 4 summarizes the factors influencing the information content with and without the SP’s demand for information.

**Table 4:** Factors influencing information content score.

	Without info demand		Total*	
	Median (IQR)	P**	Mean (SD)	P***
**Factors related to the pharmacy staff**				
**Gender**		0.04		0.62
Male	16.7 (25)		35.8 (16.7)	
Female	8.3 (25)		33.6 (15.0)	
**Age group**		0.046		0.05
20–24	18.3 (17)		31.3 (15.1)	
25–29	25 (33)		38.6 (13.3)	
30–34	18.3 (8)		22.2 (16.7)	
35–39	25 (29)		38.9 (10.1)	
≥ 40	18.3 (42)		40.3 (20.7)	
**Years of experience**	12.5 (25)	0.04	35.7 (18.1)	0.05
1–5 years	33.3 (25)		31.7 (14.9)	
6–10 years	12.5 (15)		36.0 (12.9)	
11–15 years	20.8 (29)		38.9 (12.5)	
>15 years				
**Degree**		0.02		0.10
BSc. Pharm.	18.3 (15)		32.0 (16.3)	
Pharm D	38.9 (25)		38.3 (9.5)	
Diploma	25 (33)		51.6 (3.7)	
**Confidence (1–5)**	rs = 0.194	0.16	rs = 0.32	< 0.02
**Factors related to SP visit Pharmacy type**		0.23		0.04
Independent	16.7 (33)		38.9 (16.8)	
Chain	8.3 (25)		30.4 (13.5)	
**Day of the visit**		0.33		0.07
Weekday	18.3 (25)		31.8 (14.9)	
Weekend	16.7 (33)		38.6 (16.2)	
**Time of the visit**		0.68		0.94
AM	18.3 (29)		35 (20.8)	
PM	12.5 (27)		34.5 (15.4)	
**Busyness (customers waiting)**	18.3 (33)	0.92	33.1 (16.2)	0.32
Quiet (0)	16.7 (17)		35.6 (14.2)	
Low (1–2)	18.3 (5)		47.2 (12.7)	
Moderate (3–5)				
Busy (> 5)				
**Duration (min)**	rs = 0.18	0.19	rs = 0.45	0.001
**Privacy (1–5)**	rs = 0.04	0.78	rs = 0.119	0.4
**Distraction (1–5)**	rs = 0.13	0.36	rs = 0.19	< 0.001
**SP gender**		0.03		0.18
Male	8.3 (25)		31.9 (17.7)	
Female	16.7 (25)		37.7 (12.5)	

*Total content score: content of information discussed with or without prompt or demand for information by the SP.

** As without demand information content score violated parametric test assumptions, non-parametric testing: Mann-Whitney U test, Spearman correlation, Kruskal-Wallis test and Wilcoxon were performed as appropriate.

*** As total information content score fulfilled parametric test assumptions, parametric testing: independent or paired sample T test, and ANOVA were performed.

The factors related to the pharmacy staff that scored significantly higher for information discussed spontaneously were male pharmacy staff, older age with more experience, and a Pharm D degree. When the SP prompted or demanded information, older pharmacy staff with more experience and with a college degree (i.e., pharmacy technicians) scored higher. A higher confidence in the visit was associated with better information content, particularly after the SP prompted for information. There was no significant association between confidence and other factors.

None of the factors related to the pharmacy visit were significantly associated with better information content except for the SP’s gender. However, this was not statistically significant for the total score. The SP’s gender was not statistically associated with any of the other factors.

The factors that demonstrated a significant association with the content of information discussed by the pharmacy staff spontaneously (without the SP’s demand) and the total information score (Table 4; p > 0.05) were entered into a GLM analysis. The pharmacy staff’s gender, age, years of experience degree, and the SP’s gender were entered into a spontaneous information (without the SP’s demand) score model. Similarly, for the total information score, the pharmacy staff’s age, years of experience, confidence, pharmacy type, duration of visit, and distraction were investigated via GLM. Table 5 presents the summaries of the models for the factors predicting the content of information discussed by the pharmacy staff spontaneously (without the SP’s demand) and the total information score. Levene’s tests for both models were significant (p ≤ 0.05). The homogeneity of variance was thus doubled checked via Hartley’s F max (variance ratio). The ratio of the variances between the biggest and smallest variances across groups were found below 3 (Hartley critical value). Thus, the assumption of homogeneity was deemed tenable. Homogeneity of regression slopes were checked visually and via customized modelling and showed all p values > 0.05.

**Table 5:** Model summaries of factors predicting the content of information discussed by the pharmacy staff spontaneously (without the sp’s demand) and total information score.

Parameter	B	Std. Error	t	Sig.	95% Confidence Interval	
					Lower Bound	Upper Bound
**Dependent Variable: Content of information discussed spontaneously (without the SP’s demand**)						
Intercept	1.33	0.85	1.57	0.13	-0.44	3.11
**Factors related to pharmacy staff** *						
Male pharmacy staff	0.26	0.72	0.37	0.72	-1.78	1.25
Pharmacy staff aged 20–24 years old	0.86	0.70	1.23	0.23	-2.325	0.60
Pharmacy staff aged 25–29 years old	0.76	0.62	1.24	0.23	-2.05	0.53
Pharmacy staff aged 30–34 years old	1.18	0.66	1.79	0.09	-2.558	0.20
Pharmacy staff aged 35–39 years old	0.65	0.20	0.34	**0.003**	0.06	1.24
Pharmacy staff with BSc. Pharm degree	0.64	0.48	1.326	0.20	-1.65	0.37
Pharmacy staff with Pharm D degree	0.39	0.59	0.66	0.52	-1.63	0.849
Year of experience (1–5)	0.26	0.36	0.74	0.47	-1.01	0.49
Year of experience (6–10)	0.79	0.29	2.748	**0.013**	0.19	1.40
Year of experience (11–15)	0.15	0.30	0.507	0.62	-0.78	0.48
**Factors related to SP visit** *		.	.	.	.	.
SP male gender	-0.03	0.09	-0.31	0.76	-0.213	0.16
**Dependent Variable: Total information score**						
Intercept	-0.21	0.19	-1.09	0.29	-0.61	0.19
**Factors related to pharmacy staff** **						
Pharmacy staff aged 20–24 years old	0.29	0.21	1.36	0.19	-0.15	0.73
Pharmacy staff aged 25–29 years old	0.17	0.15	1.11	0.28	-0.14	0.48
Pharmacy staff aged 30–34 years old	0.20	0.20	0.97	0.34	-0.23	0.63
Pharmacy staff aged 35–39 years old	-0.09	0.27	-0.32	0.75	-0.63	0.46
Year of experience (1–5)	0.43	0.21	2.07	**0.049**	0.003	0.85
Year of experience (6–10)	0.26	0.27	0.96	0.35	-0.29	0.80
Year of experience (11–15)	0.32	0.16	-1.92	0.07	-0.02	0.66
Confidence	0.07	0.03	2.19	0.**04**	0.004	0.13
**Factors related to SP visit** **			.	.	.	.
Independent pharmacy type	-0.04	0.12	-0.31	0.76	-0.29	0.22
Duration	0.05	0.02	2.21	**0.04**	0.003	0.09
Distraction	0.02	0.03	0.72	0.48	-0.05	0.09

*Reference (redundant) variables for factors related to pharmacy staff/SP visit: gender (female); age (≥40); years of experience (›15 years); SP’s gender (female).

**Reference variables for factors related to pharmacy staff/SP visit: age (≥40); years of experience (›15 years); pharmacy type (chain). Dummy variables for unspecified degree and year of experience were found not statistically significant.

It can be seen that pharmacy staff with older age and more years of experience were more likely to discuss information spontaneously. However, this was shown to be only significant for pharmacy staff aged 35–39 and those with 6–10 years of experience, with df 35, F = 2.24, P value = 0.032, and R squared = 0.81. After the SP demanded information, the pharmacy staff with experience less than 5 years and greater confidence as well as longer visits were positively predicting higher information scores with df 29, F = 2.96, P value = 0.007, and R squared = 0.76.

### Pharmacist views on simulated patient visits

The post visit form was not returned by one pharmacy. None of the pharmacy staff reported that they suspected the SP. All of them believed the SP acted in a natural manner (100%, n = 54), his/her appearance fit the role (94.4%, n = 51). Most that and reported the SP responded in a natural manner (96.3%, n = 52).

A majority of the pharmacy staff reported that the feedback was not confrontational (98.5%, N = 53) and was conducted in a professional manner (96.5%, N = 52). All of the pharmacy staff reported that it was easy to understand the SP and were comfortable when discussing with the SP (98.5%, N = 53).

**Fig 1:**
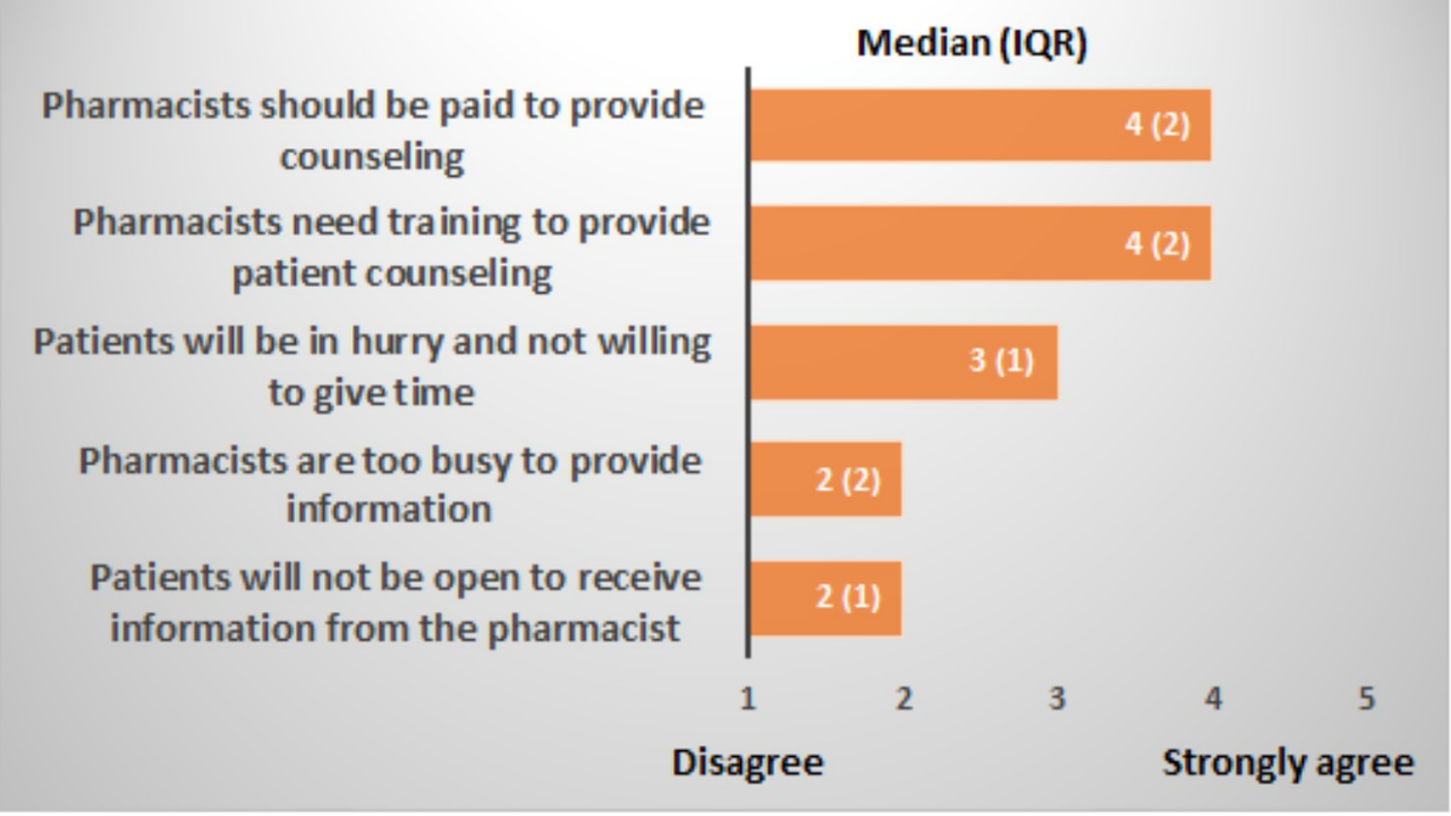
The extent of pharmacy staff agreement on: Being busy to provide information, need training, service reimbursement, or patients being open for information or in hurry in real practice.

The Median (IQR) to which the pharmacy staff agreed (1 to 5) regarding the patients’ openness to information and needs for training or reimbursement are shown in Fig 1. The pharmacy staff agreed mostly with the need for training and reimbursement.

## Discussion

This study is the first to assess community pharmacists’ management of information requests in relation to POM and contributing factors to the content of information in Jordan via the SP method. The SP approach captured a real observation of the information provisions discussed by the pharmacy staff and investigated how demand for information affected subsequent counseling content. Before the SP demanded information, only 45% of pharmacy staff discussed the indications or benefits and scientific name of the medication. For only 23% of the visits, the pharmacy staff confirmed who the medicine was for. For one third of the visits, the pharmacy staff discussed how to take it. This suboptimal practice might carry considerable risks to patient safety and health outcomes [2]. Therefore, investigating driving factors for better counseling behavior in community settings is vital to outline effective strategies to enhance practice.

Demanding or probing for information was an enabling factor for better counseling behavior. This is in line with previous studies investigating factors influencing patient participation in medication counseling at the community pharmacy [21, 25, 26]. In fact, patients not requesting information about medications have been identified as a priority medication safety problem in another setting [27].

Strategies that enhance patient involvement and promote a pharmacist-patient sharing agenda by pharmacists asking questions about the SP’s needs, checking for patient concerns or questions, and offering contact information are critical. These enhance information exchange and enable more information sharing. Skills to engage patients were only fulfilled by 10% of the visits. These deficiencies should be outlined to educators and authorities responsible for the continuous professional development of pharmacy staff especially since our findings indicate that pharmacy staff showed the highest extent of agreement with the need for training and reimbursement.

Of note, none of the pharmacy staff discussed side effects or known allergies. Even after the SP asked for information, this information was not discussed except in one visit. Reluctant to discuss such information has been reported for both OTC and POM medication [13, 21, 26]. Understanding the reasons or perspectives for such an observation is essential. This could be explored in depth via qualitative research.

Non-pharmacological advice and written communication was not common too. Previous research has shown that life style modifications can be considered a significant contributor to better disease control and progression with respect to outcomes, safety, and costs [28]. Pharmacy led services targeted to non-pharmacological advice carry professional opportunities for community pharmacists [4]. Comprehensive programs to promote and train pharmacists on these aspects are demanded [5, 29].

A key aspect of the current study is examining the relationship between the content of counseling and a number of pharmacy staff and visit characteristics. Older aged pharmacy staff with more years of experience were more likely to discuss information without patient demands. After patients demanded information, pharmacy staff with experience less than 5 years and with greater rated confidence were more likely to respond to the demand for information. Possibly, older pharmacy staff with more years of experience are more comfortable and experienced to initiate patient counseling or share information. For those who have experience less than 5 years and are potentially younger (age and year of experience are significantly associated, chi-squared test p ≤ 0.05.), they were enabled for better information sharing after the patient requested it [25]. This is an important observation to determine training needs for freshly graduated pharmacy staff and inform skill based teaching in pharmacy curriculum. Further research is needed to clarify age and experience effects and to better understand the perspectives or motivations underlining this observation.

Duration of the visit was a significant contributor to better counseling. Potentially, longer interaction time between the pharmacist and the patient would enable a better discussion of information and encourage both sides to participate in the conversation. Nevertheless, studies have shown that pharmacists have often perceived that patient counseling was time consuming and highlighted their workload as a main barrier [25, 30–32]. There is no clear cut-off time to exchange information in community settings. However, WHO outlined that three minutes are needed at least per a patient for complete counseling and orientation by the pharmacist [33]. This study demonstrates limited counseling practice in community settings regardless of the busyness or number of customers waiting. Pharmacy staff also agreed modestly that they are too busy to provide information. On the other hand, they agreed that patients are in a hurry and not willing to give time. Therefore, there might be a mismatch between the perspectives and expectations on both sides. In-depth analysis is demanded to reveal the true barriers on both sides to help form effective corrective strategies [34].

One of the strengths of this study is the use of the CRiSP checklist to improve the quality of the study methodology reporting an area that is often inadequately reported by pharmacy researchers [20]. The SPs received formal training and were evaluated before the visits to enhance authenticity and standardization. Visits were also audiotaped to minimize recall bias and human error. None of the pharmacy staff reported that they suspected the SP which confirms our confidence in the SP training. However, the study has a key limitation. The data collection included a convenient small sample of community pharmacies in Amman, the capital of Jordan. Due to the exploratory purpose of the study, a minimum sample size of 30–60 was considered tenable to support the depth of the visit-oriented analysis and the structured feedback. Future larger studies across various cities are recommended to enhance the generalizability and to enable investigation of predictive links across various levels of covariates and post hoc contrasting analysis of group effects.

## Conclusions

Community pharmacists responding to information about medications needs to be improved. A number of factors related to the pharmacy staff and the visits were found to be associated with better information exchange. The SP’s request for information facilitated more informative interaction with the patient. In-depth understanding of these factors is crucial to develop future strategies that address communication barriers, reduce passive patient encounters, and eventually contribute to patient empowerment and responsive patient-pharmacist consultations.

## Supporting information

S1 Data (SAV)
